# An Exceptional Case of Ileocolic Intussusception Secondary to Burkitt's Lymphoma: What Variations Are There in the Presentation and Management of Those Patients Who Approach Adolescence?

**DOI:** 10.1155/2018/6251321

**Published:** 2018-06-20

**Authors:** Krish Kulendran, Kay Tai Choy, Cian Keogh, Dinesh Ratnapala

**Affiliations:** ^1^Cairns Hospital, Cairns, QLD, Australia; ^2^Ipswich Hospital, Ipswich, QLD, Australia

## Abstract

Intussusception is a common cause of abdominal pain among the paediatric population with up to 10% of cases occurring secondary to a pathological lead point. Burkitt's lymphoma (BL) is a highly malignant and rapidly growing B-cell neoplasm which in extremely rare cases can present as intussusception. We report a case in an otherwise healthy 15-year-old male who presented with atypical abdominal pain. Imaging subsequently indicated an ileocolic intussusception, and given that the suspicion of a pathological lead point mandates a laparotomy and bowel resection, he proceeded to surgery. The histopathology confirmed Burkitt's lymphoma as the aetiology responsible for this intussuscepted mass. A detailed discussion including a systematic review of all previous case reports explore the diagnostic dilemma of intussusceptions secondary to BL. This case report aims to highlight the clinical challenges in establishing such a diagnosis and an appreciation for the subtle variations in clinical features, as well as the differences in management between infants and adolescents.

## 1. Introduction

In 1958, the surgeon Dennis Burkitt first observed a tumour affecting the jaw of one of the paediatric population of Equatorial Africa. This tumour was initially purported as a sarcoma of the jaw; however, it was promptly reclassified as a distinct form of non-Hodgkin's lymphoma (NHL) based on its histopathological features [1].

BL is a rapidly growing B-cell neoplasm which is highly malignant and aggressive. The variants of BL are illustrated below in Table 1.

BL is now recognized in a variety of extranodal sites, including the ileocaecal region. Here, it may cause either indirect pressure symptoms or via direct luminal involvement, intestinal obstruction, or intussusception. Complete resection of this tumour is required for optimal patient survival [3].

This case details a rare cause of intussusception secondary to Burkitt's lymphoma. In infants this is often treated with air enema reduction, but in adult populations intussusceptions are normally associated with a lead point and so surgical management is essential [4]. Identifying this diagnosis in this intervening age group is clinically challenging and a high index of suspicion is necessary. This report contains interesting diagnostic imaging, operative details, and specimen photographs.

## 2. Case Report

A healthy 15-year-old male presented with a three-week history of irretractable abdominal pain, vomiting, and anorexia. There was no previous similar history or abdominal surgery. He associated the onset of symptoms with a recent contraction of gastroenteritis within the family. There was no improvement in his condition despite his family contacts recovering.

On examination, he was afebrile and haemodynamically stable. There was a scaphoid abdomen with maximal tenderness in the right upper quadrant. There was significant guarding. Bowel sounds were audible. His abdominal X-ray and routine blood test results were both unremarkable, other than a raised C-reactive protein of 92.

**Figure 1:**
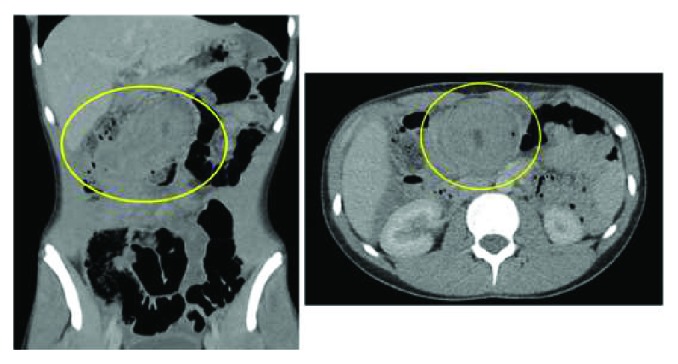
CT imaging indicating intussusception.

His high opioid requirement, chronicity of symptoms, and examination findings prompted further evaluation with CT. This revealed right-sided abdominal mass and a layering effect at the caecal pole consistent with an intussusception. The appearance was similar to a “pseudokidney,” as shown in Figure 1. There was marked free fluid within the abdominal cavity. After resuscitation, he proceeded to a laparotomy.

A diagnostic laparotomy was performed for the inspection of abdominal contents. It confirmed radiological findings of an intussusception of the terminal ileum within the caecal pole. A hard mass was noted within the hepatic flexure region. There was a dilated terminal ileum and multiple lymph nodes noted within the mesentery.

A right hemicolectomy was performed. Vascular pedicles were taken high for an appropriate oncological resection, given the suspicion. Primary ileocolic stapled side-to-side anastomosis was performed. The recovery was uncomplicated and the patient was discharged home three days postoperatively.

**Figure 2:**
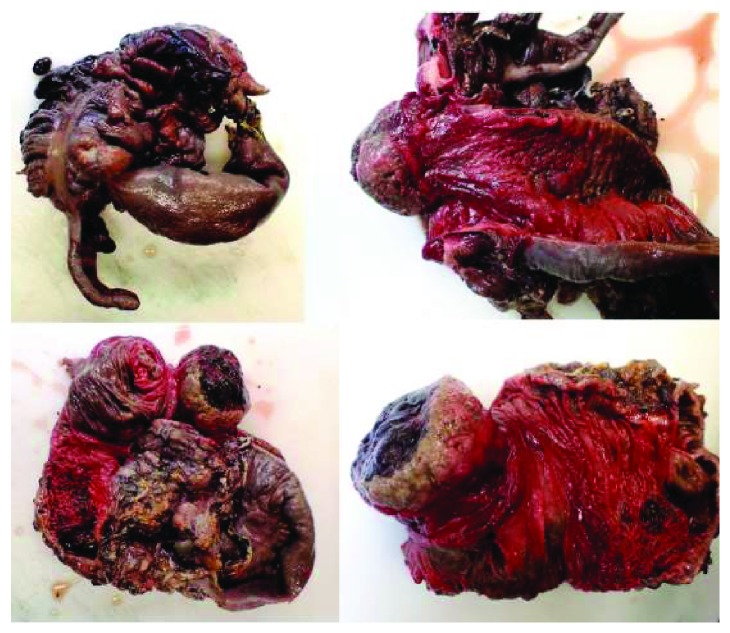
Right hemicolectomy specimen demonstrating ileocolic intussusception.

As shown in Figure 2, the histopathology of the excised mass proved to be Burkitt's lymphoma of the terminal ileum causing ileocolic intussusception. It extensively involved the appendiceal serosa, mesentery, and omentum.

**Figure 3:**
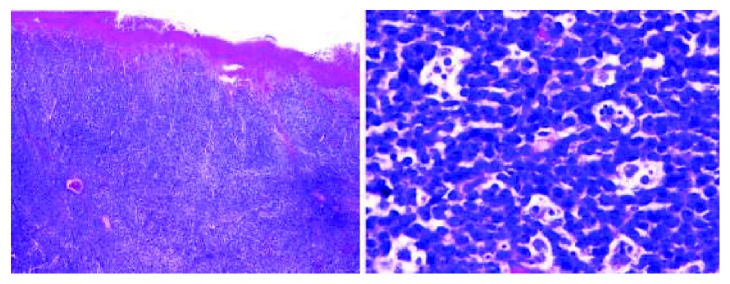
Histopathology slides demonstrating characteristic features of BL.

The tumour was an ulcerated lesion infiltrating all layers of the bowel wall. As shown in Figure 3 microscopically, the characteristic starry sky growth pattern was visualised, with the cells having round nuclei, finely clumped chromatin, and small basophilic nucleoli.

**Figure 4:**
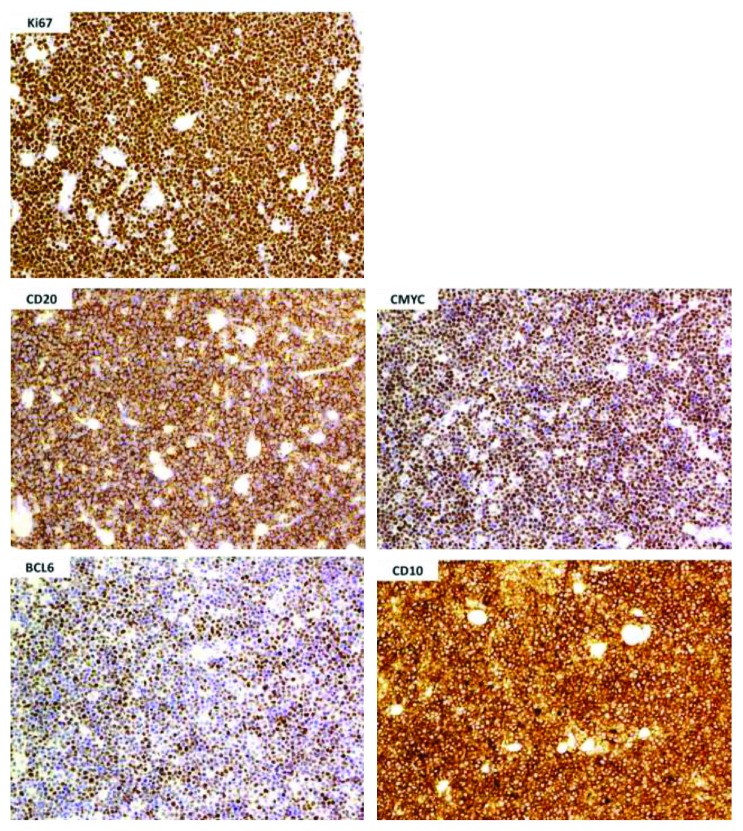
Histopathology slides demonstrating positive CD20, CD10, C-MYC, BCL6, and Ki67 immunostains.

A panel of immunostains was performed on bowel tumour, the regional lymph nodes, and the omentum. The tumour cells in all three sites are CD20, CD10, BCL6, and C-MYC positive (see Figure 4). The tumour has a very high proliferative index with almost 100% of the tumour expressing Ki67. BCL2, Cyclin D1, TdT, CD3, CD5, CD23, and CD30 are negative. Fluorescence in situ hybridisation (FISH) detected a reciprocal t(8;14) translocation and rearrangement of the C-MYC gene. As only chromosome-specific probes were used, the presence of other abnormalities cannot be excluded. The above profile supports the original diagnosis of BL.

### 2.1. Systematic Review of Relevant Literature

The medical literature in the PubMed and Medline/EMBASE databases was reviewed for cases of Burkitt's lymphoma-related intussusception. All publications were scrutinized that contained keywords of “intussusception” and “Burkitt lymphoma.” The literature search was limited to paediatric patients under 18 years old, and restricted to papers written in English.

A systematic review of relevant literature was performed to ascertain variance of clinical features in primary presentations of BL-related intussusception. The following information on patient numbers, demographic, main presenting complaint, disease stage, and diagnostic methods were obtained in this meta-analysis.

## 3. Results

A literature search was performed using the PUBMED medical database. A broad scope of analysis is ensured by including all relevant papers that fulfilled the keyword search, irrespective of age, location, or outcome measurements. Non-English papers were excluded, as time resources could not be allocated for interpretation.

The literature review identified relevant papers when searching for keywords of “Burkitt lymphoma” and “intussusception.” This yielded 31 publications reporting on 226 patients in total. However, the majority of these papers reported presentations of intussusception in known BL, rather than primary presentations. These were therefore excluded from the analysis, leaving 21 valid publications reporting on 70 paediatric patients.

The mean (s.d.) age was 5.28 (range 2.5–17) years. Sex was recorded in most cases, with a male preponderance of almost 2 : 1 distribution. A solitary case report detailed BL-related intussusception in a patient with Wiskott-Aldrich syndrome, with the remainder of the cases eliciting no significant medical background.

From our cases, the most common presenting complaint was abdominal pain, occurring in 95% of the patients. Importantly, all of these cases mentioned “recurrent” or having painful symptoms lasting >1 week. Other common symptoms included vomiting (28%), altered bowel habits (11%), and bloody stools (13%). Systemic symptoms included weight loss/malaise/fatigue in 7%. Only 13% had a palpable abdominal mass on examination. Only 1 case reported a rectal prolapse as a case of BL-related intussusception.

The investigations that were performed revealed no clear pattern—with nonspecific rises in inflammatory markers described in four cases. Every patient underwent preoperative diagnostic imaging studies—with the majority of them ultrasound/CT, which confirmed the presence of intestinal intussusception. All but one patient had ileocolic intussusception (98%), with the one exception of colocolonic intussusception at the transverse colon.

Regarding outcomes, these results were only available for 54 out of the 70 patients. Using the St. Jude staging system, the stage of disease was determined in these 54 patients. Results show that the 67% of all patients who presented with intussusception had predominantly Grades I-II disease. 36 out of the 54 patients subsequently received complete tumour resection due to the disease being limited to the area of intussusception. Complete resection was not achieved in the remaining 18 patients, of whom 12 had stage III disease (i.e., ascites, retroperitoneal nodal involvement, and liver involvement) and 6 had stage IV disease (i.e., bone marrow involvement) (Table 2).

## 4. Discussion

Primary gastrointestinal lymphoma represents 1–4% of all gastrointestinal malignancies [5], with Burkitt's lymphoma accounting for 0.3–1.3% of all non-Hodgkin's lymphomas. While BL accounts for only 1% of all adult lymphomas, it constitutes ~30% of all paediatric NHL, hence representing a significant burden of disease in this age group. As most reported cases in children affect the distal ileum/ileocaecal region, intussusception has been identified as a common presentation [5].

While the classical triad for diagnosis of intussusception comprises of abdominal colic, “red-currant jelly stools,” and palpable abdominal mass, there remains a diagnostic dilemma due to the subtler presentation of intussusception in postinfancy children older than 2-3 years where the classic triad of symptoms may not be present, such as in our case (1, 3). Among the 70 cases analysed above, only 12.8% satisfied the classical triad of intussusception at time of presentation, with the majority of cases just presenting with abdominal pain alone. However, 95% had recurrent symptoms lasting greater than a week, which highlights the importance of recognizing these atypical symptoms for intussusception.

In terms of history, previous literature have described predisposing factors for lymphoma of the small intestine including prior malabsorption syndromes, inflammatory bowel disease, and an immune-deficient state [6]. Our patient's EBV and HIV serology was checked postoperatively and found to be undetected.

Common investigations used today to workup a paediatric patient with concerning abdominal symptoms include blood tests and imaging. Common blood tests associated with an intra-abdominal neoplastic event—such as LDH, was only reported to be elevated in a small number of cases. While not elaborated in detail, most cases reported a fairly unremarkable blood panel in their patients with BL-associated intussusceptions.

Many previous case reports have described misdiagnosis at presentation with differential diagnoses including appendicitis, inflamed Meckel's diverticulum, intestinal infections, and ischaemic gut. In child-bearing females, it is essential to rule out catastrophic causes of ovarian torsion and ectopic pregnancies.

Ultrasonography has a false-negative rate approaching zero and is a reliable screening tool for children at low risk for intussusceptions. Almost 95% of the previously reported cases utilised ultrasound or computer tomography to confirm the presence of intussusception. Some consequently received a contrast enema, which is both diagnostic (the gold standard in the diagnosis of intussusception) and therapeutic [7]. Our patient's presentation and demographic was atypical of idiopathic intussusception and thus a CT was more appropriate to investigate his inexplicable pain, and identify any pathological lead point. This case highlights the importance of being aware of the differing aetiology and consequent management between young infants and adolescents.

In summary, BL-related intussusception is often challenging to diagnose, but must be considered even when facing a child who does not present with the classical triad. The suspicion of intestinal intussusception at a lead point necessitates a laparotomy. Laparotomy remains the gold standard in both diagnosis and treatment, ensuring the excision of the entire tumour with appropriate margins [8]. A multidisciplinary approach is mandatory to ensure that appropriate therapeutic management, as well as necessary follow-up, occurs [9].

This case serves as a reminder to clinicians to maintain vigilance regarding this diagnosis and the subtle variations in its presentations and management among the adolescent/paediatric population.

## Figures and Tables

**Table 1:** Describing variants of BL [2].

Variants of BL	
Type	Features
Endemic	Largely prevalent in the African continent, develops due to chromosomal translocation between chromosomes 8 and 14 causing tumours of the facial bones.
Sporadic	Nonendemic and primarily affects the abdominal viscera. It is subsequently described outside of Africa. This variant occurs due to chromosome 8 translocation involving the c-myc oncogene. This form tends to present with the lymphoid tissues of the gut. The disease can present as masses affecting the terminal ileum, caecum, and abdominal mesentery.
Immunodeficient	Frequently presents with diffuse lymphadenopathy. The Epstein-Barr and human immunodeficiency viruses are a recognized association with all forms of BL, not just the immunodeficient forms.

**Table 2:** Clinical manifestations of BL-related intussusception.

Trait	No. of patients (%)
Sex	
Male	46 (65)
Female	10 (15)
Unspecified	14 (20)
Stage of disease at diagnosis	
I	1 (2)
II	35 (65)
III	12 (22)
IV	6 (11)
Symptoms/signs	
Abdominal pain	67 (95)
Nausea/vomiting	20 (28)
Altered bowel habits	8 (11)
Blood in stools	9 (13)
Fatigue/malaise/wt. loss	5 (7)
Abdominal mass	9 (13)
Duration > 1 weeks	67 (95)
